# Matrine attenuates high‐fat diet‐induced in vivo and ox‐LDL‐induced in vitro vascular injury by regulating the PKCα/eNOS and PI3K/Akt/eNOS pathways

**DOI:** 10.1111/jcmm.14180

**Published:** 2019-02-15

**Authors:** Song Zhang, Shun Guo, Xiao‐bo Gao, An Liu, Wei Jiang, Xi Chen, Peng Yang, Lin‐na Liu, Lei Shi, Yan Zhang

**Affiliations:** ^1^ Department of Pharmacy The Second Affiliated Hospital of Air Force Medical University Xi’an PR China

**Keywords:** matrine, obesity, PI3K/Akt, PKCα, vascular endothelial injury

## Abstract

Lipid metabolism disorders lead to vascular endothelial injury. Matrine is an alkaloid that has been used to improve obesity and diabetes and for the treatment of hepatitis B. However, its effect on lipid metabolism disorders and vascular injury is unclear. Here, we investigated the effect of matrine on high‐fat diet fed mice and oxidized low‐density lipoprotein (ox‐LDL)‐induced human umbilical vein endothelial cells (HUVECs). Computational virtual docking analyses, phosphoinositide 3‐kinase (PI3K) and protein kinase C‐α (PKCα) inhibitors were used to localize matrine in vascular injuries. The results showed that matrine‐treated mice were more resistant to abnormal lipid metabolism and inflammation than vehicle‐treated mice and exhibited significantly alleviated ox‐LDL‐stimulated dysfunction of HUVECs, restored diminished nitric oxide release, decreased reactive oxygen species generation and increased expression phosphorylation of AKT‐Ser473 and endothelial nitric oxide synthase (eNOS)‐Ser1177. Matrine not only up‐regulates eNOS‐Ser1177 but also down‐regulates eNOS‐Thr495, a PKCα‐controlled negative regulator of eNOS. Using computational virtual docking analyses and biochemical assays, matrine was also shown to influence eNOS/NO via PKCα inhibition. Moreover, the protective effects of matrine were significantly abolished by the simultaneous application of PKCα and the PI3K inhibitor. Matrine may thus be potentially employed as a novel therapeutic strategy against high‐fat diet‐induced vascular injury.

## 1 INTRODUCTION

With the rapid development of the global economy and improvements in lifestyle, the overweight and obesity epidemic has become a major health challenge around the world.^1^ High‐fat diets play a primary role in obesity and can increase the risk for various diseases such as type 2 diabetes mellitus, cardiovascular diseases and other metabolic diseases.^2^, ^3^, ^4^ Vascular endothelial dysfunction is closely related to cardiovascular diseases. Vascular endothelial cells play a key role in maintaining vascular permeability, transmitting vascular information and secreting vasoactive substances. Vascular endothelial cell injury plays an important role in the pathological process of atherosclerosis, hypertension, diabetes, cerebrovascular disease and other pathological processes.^5^, ^6^, ^7^, ^8^ Obesity can lead to disorders of lipid metabolism, which are characterized by an increase in the rates of triglyceride (TG), total cholesterol (TC) and low‐density lipoprotein (LDL) and a reduction in high‐density lipoproteins (HDL). Lipid metabolism disorders can contribute to a reduction in the production and/or bioavailability of nitric oxide (NO), an important endothelium‐dependent relaxant factor, thereby leading to vascular dysfunction. Endothelial nitric oxide synthase (eNOS)/NO plays a key role in the pathogenesis of abnormal lipid metabolism‐induced cardiovascular complications.^9^, ^10^ Therefore, in recent years, the eNOS/NO pathway has been considered as a critical target for mediating endothelial function.

Matrine (Mat), a tetracyclo‐quinolizidine alkaloid that is mainly derived from leguminosae such as *Sophora flavescens* and *S subprostrata,* has been shown to possess diverse pharmacological activities. In Asia, *S flavescens* and *S subprostrata* are commonly used in meat soups and are thought to improve obesity and diabetes.^11^ Mat has been widely used in the clinic for the treatment hepatitis B and also has exhibited a number of therapeutic effects on cardiovascular diseases.^12^, ^13^ Mat can protect cardiomyocytes from ischemia/reperfusion injury and also can improve isoproterenol‐induced myocardial injury via regulating nitric oxide synthase.^14^, ^15^ However, the mechanisms of Mat in endothelial vascular injury due to lipid metabolism disorders have not been studied. Furthermore, details on the molecular mechanism underlying the cardiovascular protective effect of Mat are limited. Thus, the present study explored the possible molecular pathways of Mat in relation to its cardiovascular protective effects.

## 2 MATERIALS AND METHODS

### 2.1 Materials

Mat (C_15_H_24_N_2_O; purity >98%) was purchased from Sigma (St. Louis, MO, USA). A high‐fat diet (HFD‐TP26301, 60 kcal% fat) was purchased from Trophic Animal Feed High‐tech Co., Ltd. (Jiangsu, China). TC, TG, tumour necrosis factor alpha (TNF‐α), interleukin‐6 (IL‐6), interleukin‐10 (IL‐10), methylthiazolyldiphenyl‐tetrazolium bromide (MTT), lactate dehydrogenase (LDH), reactive oxygen species (ROS), endothelial nitric oxide synthase (eNOS), NO and Hoechst 33258 detection kits were purchased from Beyotime Biotech Co. (Shanghai, China). The protein kinase C (PKC) activity assay kit (Abcam, UK), phosphoinositide 3‐kinase (PI3K) inhibitor: LY294002, eNOS inhibitor: nitro‐L‐arginine methyl ester (L‐NAME) and PKCα inhibitor: Go6976 were purchased from MedChemExpress Co. (Shanghai, China). The antibodies included anti‐AKT (phospho Ser473), anti‐PKCα (#4060, #2056; Cell Signaling Technology, USA), anti‐phosphorylated PKC‐α (sc‐377565; Sant Cruz Biotechnology, USA), anti‐Akt (ab8805; Abcam), anti‐eNOS (phospho Ser1177, thr945) (#9570, #9574; Cell Signaling Technology), anti‐eNOS (ab76198; Abcam) and GAPDH (AT0002; CMCTAG, USA). All other chemicals and solutions were of the highest quality available commercially.

### 2.2 Experimental animals

Male C57BL/6 mice (weight range: 16‐18 g) were purchased from the animal centre of the Fourth Military University (Xi'an, China) and housed in a controlled environment (22 ± 2°C, 12 hours light/dark cycle, free access to food and water). The mice were fasted for 12 hours before experimentation. All experiments were conducted between 8:00 am and 13:00 pm in a quiet room with temperature of 22‐24°C. All procedures involving animals and their care were conducted in conformity with the NIH guidelines (NIH Pub. No. 85‐23, revised 1996) and were approved by the Fourth Military University committee on animal care and use.

### 2.3 Experimental design

After 2 weeks of adaptive rearing, the mice were randomly divided into five groups: a control group (CON, n = 10), high‐fat diet group (HFD, n = 10) and a high‐fat diet combined with Mat (0.5, 2.5, 10 mg/kg) intervention group [HFD+Mat low (L), medium (M) and high (H) dose, respectively, n = 10]. The control group was fed with a normal chow diet and the HFD groups were given the high‐fat diet for 12 weeks. Mat was added from 5 to 12 weeks at different concentrations once daily and at the same time. Body weights were monitored every 2 weeks. At the end of the experiment, all mice were fasted for 12 hours, then anaesthetized for blood collection and killed to collect the aorta. Blood samples were centrifuged at 1000 *g* for 10 minutes at 4°C to isolate the sera.

### 2.4 Biochemical analyses

Triglyceride, TC, LDL and HDL levels were measured using an automatic biochemical analyzer (200FR; Toshiba, Japan). Pro‐inflammatory cytokines (TNF‐α, IL‐6 and IL‐10) and NO levels in the serum were assessed with commercial kits based on the colorimetric method, followed the manufacturer's recommendations and were performed in triplicate.

### 2.5 Histological examination

Each aorta, which was obtained after decapitation of each mouse, was washed in saline and fixed in 10% formalin for routine haematoxylin and eosin (H&E) staining and histopathological examination. The fixed tissues were processed routinely, embedded in paraffin wax, sectioned into 5‐μm‐thick sections in a rotary microtome and then stained with H&E dye. At least three different sections were examined per aorta sample.

### 2.6 Cell culture

Human umbilical vein endothelium cells lines (HUVECs) were a kind gift from Professor Wei Zhang of the Fourth Military Medical University. HUVECs were cultured in DMEM/high glucose medium containing 10% foetal bovine serum, 100 U/mL penicillin and 100 U/mL streptomycin and were incubated at 37°C in 5% CO_2._ When the cells had grown to about 80% confluency, the concentration of ox‐LDL (100 μg/mL) was added to the medium and the cells were then cultured for another 24 hours.^16^, ^17^

### 2.7 Assessment of cell viability and LDH levels

Human umbilical vein endothelial cells at the logarithmic growth phase were cultured in 96‐well plates at a density of 5 × 10^4^ cell/mL and were incubated for 24 hours and then treated as follows: (a) 5, 20 and 80 μmol/L Mat for 12 hours, followed by ox‐LDL for 24 hours; (b) PI3K inhibitor 10 μmol/L (LY‐294002; Sigma, USA) and eNOS inhibitor 100 μmol/L (L‐NAME, Sigma, USA) were added 30 minutes before the addition of Mat, followed by ox‐LDL and incubation for 24 hours; then, 20 μL MTT (5 mg/mL) was added to each well followed by incubation for another 4 hours. Finally, the culture medium containing the MTT solution was removed and the resulting formazan crystals were dissolved in 100 μL of dimethylsulphoxide solvent (DMSO). The absorbance of each well was measured at a wavelength of 546 nm using a microplate absorbance reader (Tecan infinite M200 Pro, Switzerland). ox‐LDL‐induced HUVEC injury was also assessed based on LDH release into the culture medium. The cell culture medium was collected for the quantification of LDH in the supernatant according to the supplier's instructions^18^; the culture medium was collected and centrifuged. Next, the supernatant of each sample was transferred into a 96‐well plate, incubated with the LDH detection substrate mix that was added to each well and the absorbance was read at a wavelength of 450 nm on a microplate absorbance reader (Tecan infinite M200 Pro, Switzerland).

### 2.8 Measurement of ROS and NO levels

The cells were treated with 80 μmol/L Mat for 12 hours and then with 100 μg/mL ox‐LDL for 24 hours, with or without pretreatment with LY‐294002 and L‐NAME. Then, intracellular ROS and NO levels were detected as previously described.^19^, ^20^ After the indicated treatment, the cells were washed three times with PBS and were then incubated with 10 mmol/L 21,71‐dichlorofluorescin diacetate (DCFH‐DA) in serum‐free medium at 37°C for 30 minutes. DCFH fluorescence was measured using an OLYMPUS IX53 fluorescence microscope (Olympus, Tokyo, Japan) at an excitation wavelength of 488 nm and an emission wavelength of 525 nm. The results were normalized to the fluorescence intensity of the control group. The NO levels were measured using a 4‐amino‐5‐methylamino‐2,7‐difluorofluorescein diacetate (DAF‐FM DA) fluorescence probe kit. Using a fluorescence spectrophotometer (Tecan Infinite M200 Pro, Switzerland), fluorescence intensities were measured at an excitation wavelength of 495 nm and an emission wavelength of 515 nm.

### 2.9 Measurement of PKC and eNOS activity

Cells from different groups were washed twice with PBS and scraped, followed by the addition of ATP to initiate a kinase reaction and were then incubated for 90 minutes at 30°C. Subsequently, a phosphor‐specific antibody was added to each well, incubated for 60 minutes at room temperature, followed by the addition of an HRP‐conjugated secondary for 30 minutes. The optical density of each well was measured at a wavelength of 450 nm and was used in the determination of PKC activity as previously described.^21^ The reactions were performed individually in the presence of phospholipids (activated PKC reaction) and in the absence of phospholipids (control reaction). eNOS activity was measured using a NO synthase assay kit (Beyotime Biotech, China) according to the manufacturer's instructions.^22^ Cells were resuspended in 100 μL NOS detection buffer and 100 μL reaction buffer containing NOS substrates (5 μL each of 0.1 mmol/L NADPH and L‐arginine) and NO fluorescent probe resuspension (200 μL of 5 μmol/L DAF‐FM DA) and were incubated at 37°C in the dark for 30 min. When the amount of inducible nitric oxide synthase (iNOS) was determined, an inhibitor (1 mmol/L EGTA, iNOS Ca^2+^ independent, eNOS Ca^2+^ dependent, EGTA can complex Ca^2+^ in samples) was added. The reaction was terminated after 30 minutes at 37°C. Using a fluorescence spectrophotometer (Tecan Infinite M200 Pro, Switzerland), fluorescence intensities were measured at an excitation wavelength of 495 nm and an emission wavelength of 515 nm to determine total NOS (tNOS) and iNOS activity. eNOS activity was calculated by subtracting iNOS from tNOS.

### 2.10 Apoptosis assay

Cells from different groups were digested with trypsin without EDTA, resuspended in calcium‐enriched buffer, stained with Annexin V‐FITC for 5 minutes and PI for 15 minutes and then analysed by flow cytometry (Novocyte 2040R, ACEA, USA). Cell apoptosis was also determined with a Hoechst 33258 kit according to the manufacturer's instructions. The cells were observed under a fluorescence microscope (Olympus) and Hoechst 33258 was detected at an excitation wavelength of 340 nm. For each well, three visual fields were randomly selected. The number of positive‐staining HUVECs and total cells were counted and apoptosis was evaluated by the ratio of positively stained cells to the total number of HUVECs.

### 2.11 Western blot analyses

Total protein was isolated from cultured HUVECs cells with RIPA lysis buffer and protein concentrations were measured using a BCA kit (P0012; Beyotime Biotechnology, China) in accordance with the manufacturer's instructions. For the determination of protein levels, total proteins from each sample (50 μg) were separated by 10% sodium dodecyl sulphate polyacrylamide gel electrophoresis (Bio‐Rad, CA, USA) and were electrotransferred onto the nitrocellulose (NC) membranes. The NC membranes were blocked with 5% non‐fat dry milk in Tris‐buffered saline containing 0.05% Tween 20 (TBST) for 1 hour at room temperature and were then incubated with anti‐Akt, anti‐Akt ser473, anti‐eNOS, anti‐eNOS ser1177, anti‐eNOS thr495, anti‐PKCα, antiphospho‐PKCα (1:1000) and anti‐GAPDH (1:3000) primary antibodies overnight at 4°C. Then, the membranes were washed four times for 10 minutes each with TBST buffer; the appropriate secondary antibody conjugated with HRP (1:5000) was used to tag the primary antibody for 2 hours. The membranes were washed three times with TBST (10 minutes each time). The immunoreactive bands were visualized using an enhanced chemiluminescence detection system (Millipore immobilonTM HRP Substrate). The immunoreactive bands on the autoradiography films were scanned with a calibrated densitometer, ChemiDoc^TM^ XRS+ (Bio‐Rad imaging System) and were quantified using QuantityOne imaging software (Bio‐Rad Laboratories, Hercules, CA, USA). Equal amounts of protein were loaded onto the gel and were confirmed by immunodetection of GAPDH.

### 2.12 Molecular docking studies

The docking studies were conducted using AutoDock Vina software. The input files were prepared in the graphic interface AutoDock Tools 1.5.2.^23^ All the ligands in the computational study were converted into 3D format. The docking parameters were as follows: blind docking procedure with a grid box of 126 × 126 × 126 Å^3^ and a grid spacing of 0.375 Å. For scoring, we selected the Lamarckian Genetic Algorithm with maximum number of energy evaluations of 1 × 10^7^ among the entire cluster of complexes for PKCθ (PDB code: 1XJD) and PKCα (PDB code: 4RA4), predicted by Autodock.

### 2.13 Statistical analyses

The statistical analyses of the data to determine significant variations among groups was performed with SPSS 17.0 statistical software. The data were expressed as the mean ± SD. Multiple comparisons between groups were performed with one‐way ANOVA and pairwise comparisons were conducted by independent *t* test. In all cases, differences with a *P < *0.05 were considered statistically significant.

## 3 RESULTS

### 3.1 Mat improves lipid metabolism and inflammation in HFD mice

**Figure 1:**
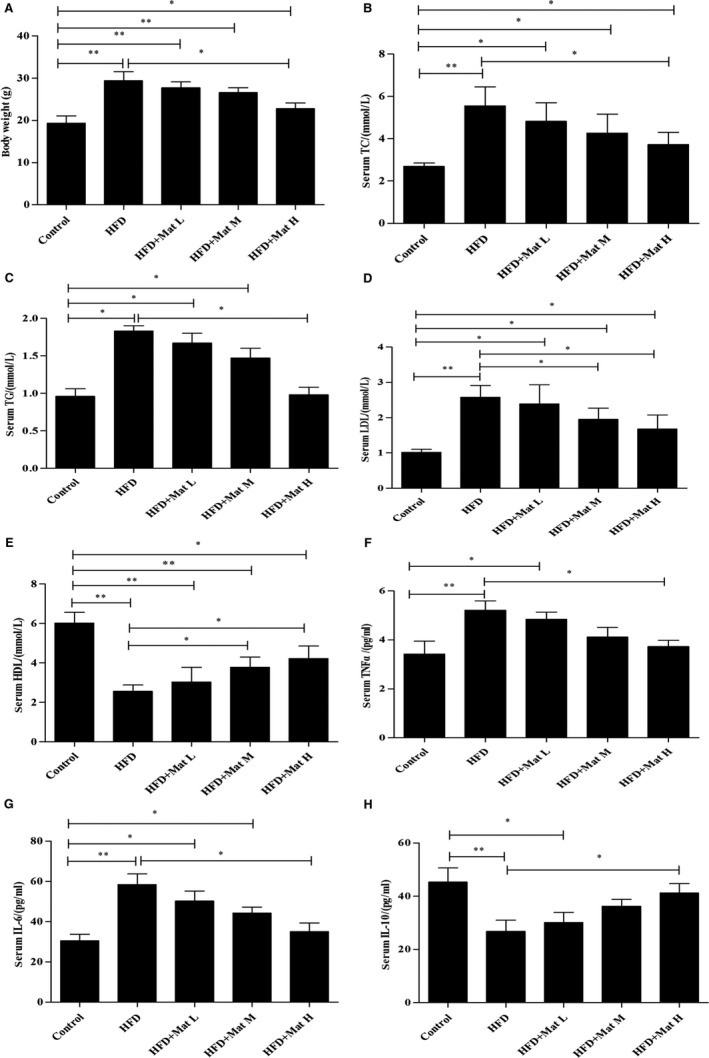
Matrine improves lipid metabolism and inflammation in HFD mice. A, Body weight of C57BL/6 mice from all groups after 12 wk on an HFD. B‐E, Serum biochemical parameters of TC, TG, LDL and HDL in all groups after 12 wk on an HFD. F‐H, Serum levels of inflammatory factors of TNF‐α, IL‐6 and IL‐10 in all groups after 12 wk on an HFD. Data are expressed as the mean ± SD (n = 10 each group). *P < 0.05, **P < 0.01

At baseline, no significant differences in body weight were observed among all groups (data not shown). The weight of the mice that were fed the HFD significantly increased (the weight of mice in the fourth and eighth weeks was measured; the results are presented as supplemental data). After 12 weeks of feeding, the body weight of the HFD mice significantly increased compared to those on a normal diet (*P* < 0.05). Mat administration reduced the HFD‐induced body weight gain in a dose‐dependent manner (Figure 1A). In addition, the mice fed with HFD showed significantly increased serum TC, TG and LDL levels, whereas that of HDL significantly decreased compared with those on the normal diet (*P* < 0.05). The changes in TG, TC, LDL and HDL levels in the HFD‐fed mice were effectively alleviated by Mat treatment (Figure 1B‐E). Furthermore, the application of high‐dose Mat resulted in a significant reduction in the TG content, reaching a similar level as that in the control mice. The serum levels of inflammatory factors were also measured. Compared with the mice fed the normal diet, serum levels of TNF‐α and IL‐6 significantly increased, whereas that of IL‐10 decreased in mice fed the HFD. Furthermore, serum levels of inflammatory factors TNF‐α, IL‐6 significantly decreased when pretreated with Mat. IL‐10 increased after Mat treatment in mice fed with HFD and even showed no significant difference compared with the control mice when pretreated with high dosage of Mat (Figure 1F‐H).

**Figure 2:**
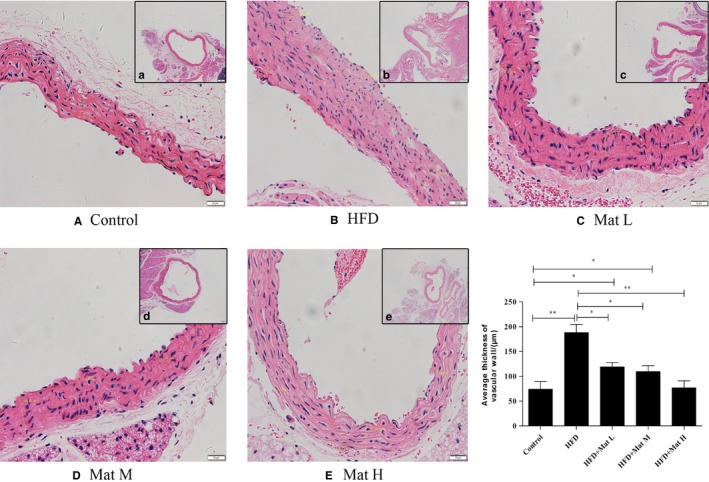
Representative photomicrographs of vascular walls were stained with haematoxylin‐eosin for histological evaluation. A, Control group. B, High‐fat diet group. C‐E, High‐fat diet combined with matrine from low to high dosages, n = 10 each group (Original magnification: a‐e, 40× objective, scale bar: 200 μm, A‐E, 200× objective, scale bar: 50 μm)

Histological analyses showed that the HFD‐treated mice exhibited a significant increase in the thickness of the aortic wall and lymphocytic infiltrates compared to those in the normal diet group (*P* < 0.01), whereas Mat effectively improved the thickening of the aortic wall and lymphocytic infiltration compared with the HFD group (*P* < 0.01 and *P* < 0.05). The average vascular wall thicknesses of each group were as follows: control group, 73.75 μm; high‐fat diet group, 187.92 μm; HFD combined with Mat from low to high dose, 118.94, 109.45 and 76.87 μm. No significant difference in the average vascular wall thicknesses between the mice fed with high‐dose matrine and normal mice was observed (Figure 2).

### 3.2 Effects of Mat on ox‐LDL‐induced HUVEC injury

**Figure 3:**
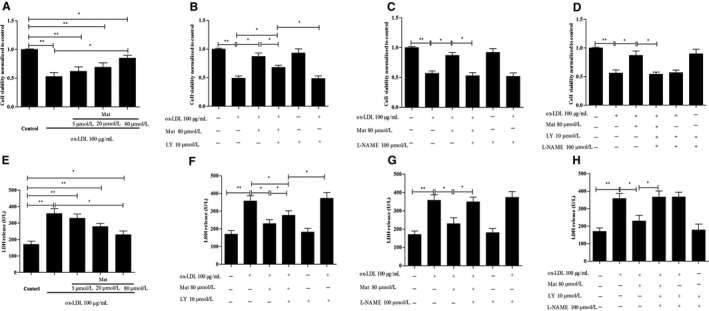
Effects of matrine on ox‐LDL‐induced HUVEC injury. Approximately 5 × 104 cells/mL were subjected to MTT and LDH assays for cell viability assessment. A and E, Quantification of the cell viability of HUVECs exposed to ox‐LDL with or without matrine pretreatment, n = 3 from three independent experiments. B‐D and F‐H, Quantification for the cell viability of HUVECs exposed to ox‐LDL along with matrine and/or LY‐294002/L‐NAME. n = 3 from three independent experiments; cell viability was normalized to the control group. Data are shown as the mean ± SD, *P < 0.05, **P < 0.01

The cell viability of HUVECs that were exposed to 100 μg/mL ox‐LDL for 24 hours significantly decreased compared to the control cells. Mat pretreatment effectively alleviated ox‐LDL‐induced cell injury in a dose‐dependent manner, increasing cell viability as determined by MTT (Figure 3A). On the contrary, when combined with LY‐294002, this protective effect significantly decreased compared to the matrine‐only group, but could still reflect the protective effect in ox‐LDL‐exposed HUVECs (Figure 3B). However, when Mat was combined with the L‐NAME (eNOS inhibitor), its protective effects were completely counteracted (Figure 3C and D). The LDH release levels of HUVECs incubated with ox‐LDL significantly increased compared to those of vehicle‐treated HUVECs, whereas those subjected to Mat pretreatment showed a significant decrease (Figure 3E). Interestingly, LY‐294002 co‐treatment did not completely abolish such protective effects of Mat. In contrast, L‐NAME combined with Mat completely diminished the protective effects of Mat (Figure 3F‐H).

### 3.3 Effects of Mat on ox‐LDL‐induced apoptosis in HUVECs

**Figure 4:**
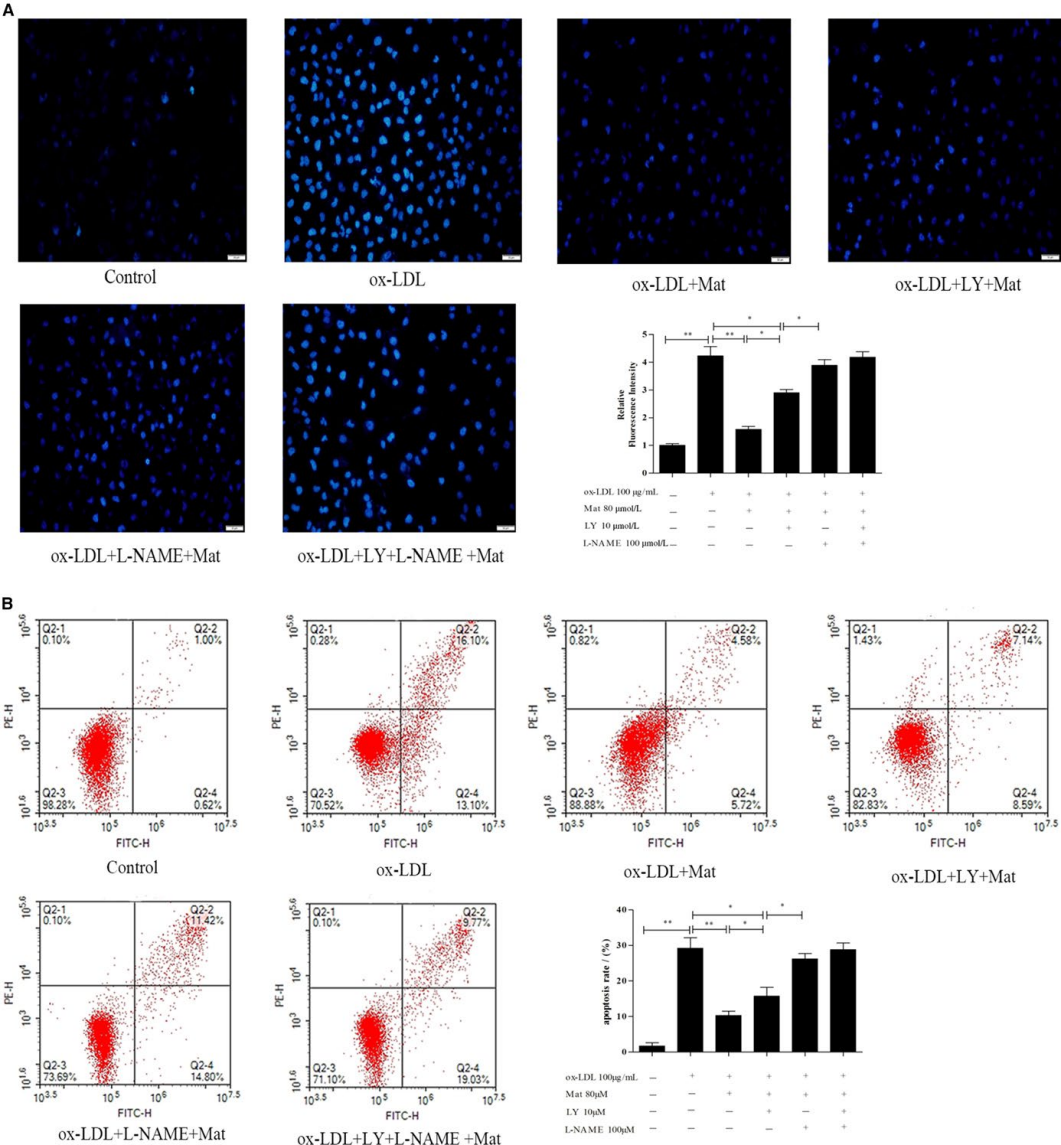
Effects of matrine on ox‐LDL‐induced apoptosis in HUVECs. A, Hoechst 33258 staining in cultured HUVECs, fluorescence intensities were measured using a fluorescence microscope; representative fluorescence images are shown (100×). B, Apoptosis analysed using Annexin V‐FITC/PI staining in a flow cytometry assay. Representative images of cell populations are shown and quantitative assessment of three independent cell apoptosis experiments was performed. Three independent samples were used. Data are shown as the mean ± SD, *P < 0.05, **P < 0.01

Apoptotic cells were detected by Hoechst 33258 and flow cytometry based on Annexin V‐FITC/PI double attaining. The Hoechst 33258 staining results showed that ox‐LDL dramatically increased the number of apoptotic cells compared to the vehicle control (*P* < 0.01) (Figure 4A). Pretreatment of HUVECs with Mat significantly reduced the rate of ox‐LDL‐induced apoptosis, whereas Mat co‐treatment with LY‐294002 resulted in a significant decrease in this protected effect compared to the Mat‐only group, but also showed a protective effect against apoptosis compared to the ox‐LDL group. In the Mat co‐treatment with L‐NAME, the protective effect disappeared, whereas Mat combined with LY‐294002 and L‐NAME showed the same effect as the L‐NAME‐only treatment (Figure 4A) Moreover, Annexin V‐FITC/PI double staining also showed similar results (Figure 4B).

### 3.4 Effects of Mat on NO and ROS in ox‐LDL‐exposed HUVECs

**Figure 5:**
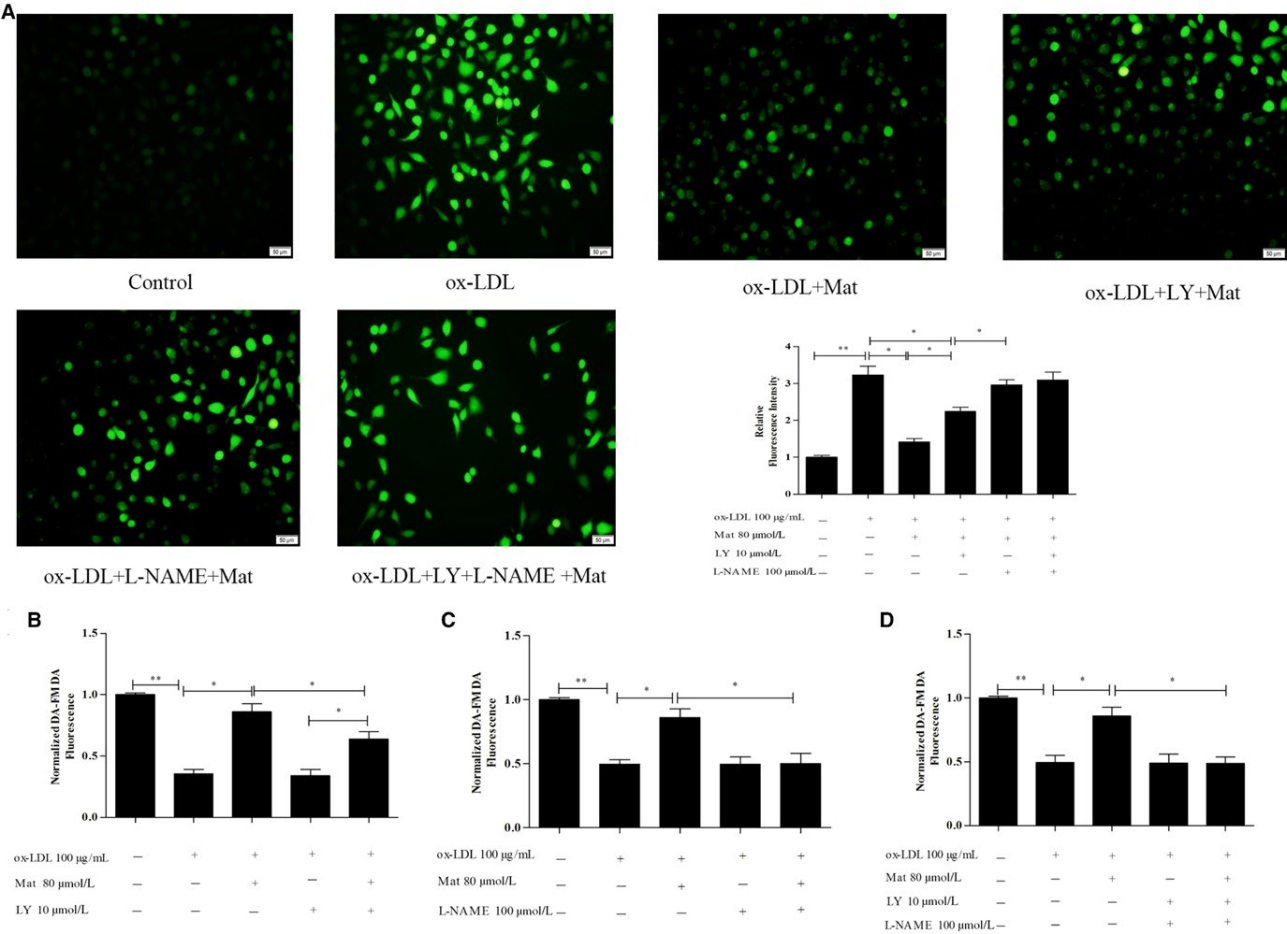
Effects of matrine on ROS generation and nitric oxide (NO) production in ox‐LDL‐exposed HUVECs. A, Intracellular ROS levels were detected using the probe DCFH‐DA; fluorescence intensities were measured by fluorescence microscopy; representative fluorescence images are shown (100×). NO levels were measured with the probe DAF‐FM DA; fluorescence intensities were measured by fluorescence spectrophotometry and untreated cells were assigned a value of 100%. B, HUVECs exposed to ox‐LDL with or without matrine pretreatment and LY‐294002. C, HUVECs exposed to ox‐LDL with or without matrine pretreatment and L‐NAME (D) HUVECs exposed to ox‐LDL with matrine, LY‐294002 and L‐NAME. Three independent samples were used. Data are expressed as the mean ± SD, *P < 0.05, **P < 0.01

The ROS levels in ox‐LDL‐exposed HUVECs significantly increased and was counteracted by Mat pretreatment. Figure 5A shows that LY‐294002 could not completely block the ROS reduction effect of Mat, but L‐NAME co‐treatment completely prevented this protective effect, whereas Mat combined with LY‐294002 and L‐NAME induced the same effect as that of L‐NAME‐only. The NO in the ox‐LDL‐exposed HUVECs significantly dropped relative to the control HUVECs, whereas Mat pretreatment resulted in a significant increase. LY‐294002 co‐treatment did not completely prevent the protective effect of Mat, but when L‐NAME was combined with Mat, the NO content did not increase in ox‐LDL‐exposed HUVECs and Mat combined with LY‐294002 and L‐NAME induced the same effect as that using L‐NAME alone (Figure 5B‐D).

### 3.5 Effects of Mat on eNOS in ox‐LDL‐exposed HUVECs

**Figure 6:**
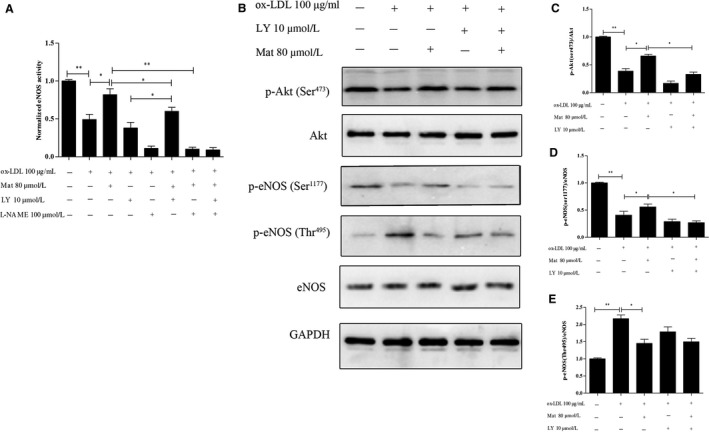
Effects of matrine on eNOS activity and PI3K/Akt/eNOS pathway‐related protein expression in ox‐LDL exposed HUVECs. A, Changes in eNOS activity in HUVECs exposed to ox‐LDL with or without matrine pretreatment along with matrine and/or LY‐294002/L‐NAME as measured using an eNOS activity assay kit. B‐E, Phosphorylation levels of Ser473Akt, Ser1177eNOS, Thr495eNOS, total Akt and the eNOS protein were measured by Western blotting. Representative images of three experiments; densitometric analysis of phosphorylated proteins was normalized to that of total proteins. Data are expressed as the mean ± SD of three independent experiments, *P < 0.05, **P < 0.01

Endothelial nitric oxide synthase activity in HUVECs treated with ox‐LDL significantly decreased compared to the control HUVECs (*P* < 0.05). Mat pretreatment effectively increased eNOS activity in ox‐LDL‐exposed cells (*P* < 0.05). LY‐294002 co‐treatment did not completely abolish this increase in eNOS activity induced by Mat. However, we also observed that Mat combined with L‐NAME did not increase the activity of eNOS, whereas Mat combined with LY‐294002 and L‐NAME had the same effect as that of L‐NAME alone (Figure 6A).

The PI3K/Akt/eNOS pathway plays an important role in the regulation of NO production and it has been shown that activated Akt phosphorylates eNOS at Ser1177, stimulates eNOS activity and induces NO release.^24^, ^25^ Furthermore, the activity of eNOS is influenced not only by the phosphorylation of eNOS‐Ser1177 but also by the phosphorylation of eNOS‐Thr495 and thus we also assessed the phosphorylation eNOS‐Thr495. In addition, ox‐LDL prominently repressed the phosphorylation of Ser473Akt and Ser1177eNOS. Mat pretreatment effectively reduced the inhibitory effect on the phosphorylation of Ser473Akt and Ser1177eNOS in ox‐LDL‐induced HUVECs. However, when Mat was combined with LY‐294002, the regulatory effect on Ser473Akt and ser1177eNOS was abrogated. ox‐LDL also significantly increased the phosphorylation of Thr495eNOS and interestingly, Mat could also significantly repress the phosphorylation of Thr495eNOS, even when combined with LY294002 (Figure 6B‐E). These data suggest that Mat rescues the ox‐LDL‐induced inhibition of NO production not only via activation of the PI3K/Akt/eNOS pathway in HUVECs.

### 3.6 Mat has significant affinity for PKCα, but not PKCθ

**Figure 7:**
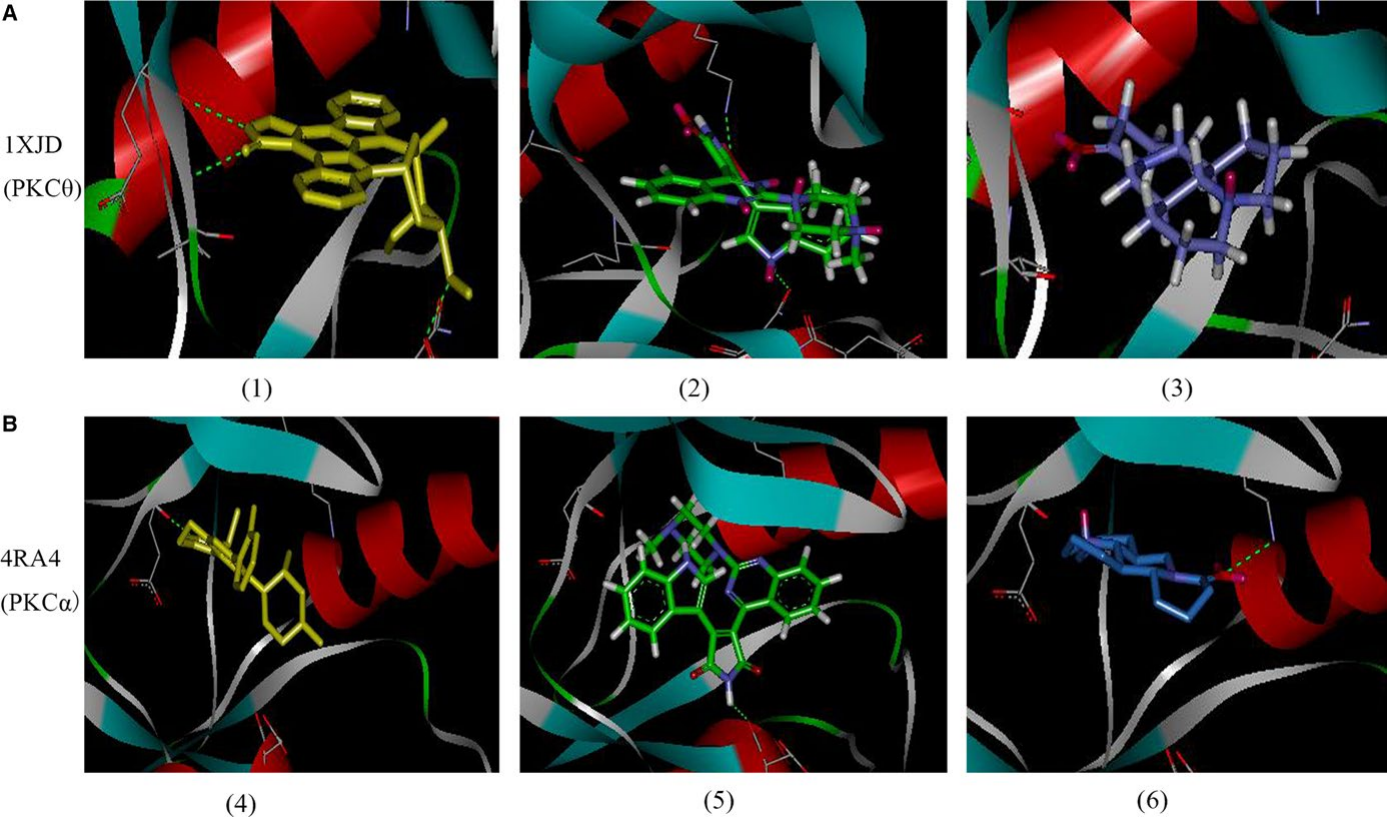
Computational docking of 1XJD (PKCθ) and 4RA4 (PKCα) with matrine. (A 1‐3) stick model, virtual ligand in PKCθ (depicted in coloured yellow), sotrastaurin in PKCθ (depicted in coloured green), matrine in PKCθ (depicted in coloured blue). (B 4‐6) stick model, virtual ligand in PKCα (depicted in coloured yellow), sotrastaurin in PKCα (depicted in coloured green), matrine in PKCα (depicted in coloured blue)

The PKC pathway also participates in the regulation of eNOS. Computational virtual ligand docking studies were performed to evaluate the molecular interactions between Mat with PKCα or PKCθ. Mat does not combine with 1XJD (PKCθ), only forms non‐polar bonds with ALA407, LEU511, ALA521, VAL394 and ALA407 in the binding pocket and does not form H bonds (Figure 7A). Sotrastaurin, a PKCθ and PKCα inhibitor, is relatively better than Mat. H bonds are formed with ASP465, LYS409 and ASN509, which greatly enhances binding to PKCθ. When Mat was combined with PKCα, the carbonyl group formed H bonds with the Lys368 amino group, thereby enhancing intermolecular forces. At the same time, it can form non‐polar interactions with VAL420, ALA480, VAL353, LYS368 and ALA366 (Figure 7B). When sotrastaurin is combined with PKCα, the N atoms on the pyrrole ring can form H bonds with the amino groups of ASP467, thereby enhancing the intermolecular interaction force. At the same time, it can form non‐polar interactions with MET470, ALA480, VAL353, MET417 and ALA366.

### 3.7 Effects of Mat on regulating PKC activity, PKCα, eNOS phosphorylation and NO content in ox‐LDL‐exposed HUVECs

**Figure 8:**
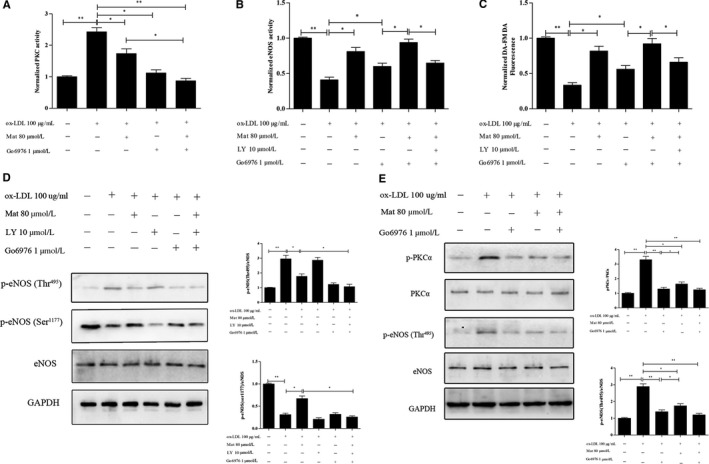
Effects of matrine on PKC and eNOS activity, eNOS phosphorylation and nitric oxide (NO) content in ox‐LDL exposed HUVECs. A, Changes in PKC activity in HUVECs exposed to ox‐LDL with or without matrine pretreatment along with matrine and/or Go6976 as measured using PKC activity assay kits. B and C, Changes in eNOS activity and NO content in HUVECs exposed to ox‐LDL with or without matrine pretreatment along with matrine and/or LY‐294002/Go6976 measured using eNOS activity assay kits and the (DAF‐FM DA) probe for the NO content. D and E, Phosphorylation levels of PKCα, Thr495eNOS, Ser1177eNOS and the total eNOS protein measured by Western blotting. Three independent samples were used. The data are expressed as the mean ± SD, *P < 0.05, **P < 0.01

Phosphorylation of Thr495eNOS is PKC‐dependent; activation of PKC results in the phosphorylation of eNOS at Thr495. The PKC activity of HUVECs treated with ox‐LDL significantly increased compared to the control HUVECs (*P* < 0.05), whereas Mat pretreatment effectively decreased PKC activity in ox‐LDL‐exposed HUVECs (*P* < 0.05) (Figure 8A). The inhibition effect is similar to that of the PKCα inhibitor Go6976. The PKCα inhibition effect using Mat combined with Go6976 was not significantly different from that using Go6976 alone. Pretreatment with the PKCα inhibitor Go6976 increased eNOS activity and the NO content in ox‐LDL induced HUVECs and this regulatory effect on eNOS activity and NO further improved when Go6976 was combined with Mat. It is important to note that when Mat, LY‐294002 and Go6976 were given simultaneously, the regulatory effect was not significantly different from treatment with Go6976 alone (Figure 8B and C).

Furthermore, Western blotting results showed that Mat could significantly reduce the phosphorylation of Ser657PKCα, which is similar to the effect of PKCα inhibitors. This regulatory effect on Ser657PKCα did significantly differ between Go6976 alone and when matrine was combined with Go6976. Mat blocked the phosphorylation Thr495eNOS, which is similar to the effect of PKC inhibitors. Mat combined with LY294002 had no significant effect on the regulatory effect. These results indicated that Mat blocks the phosphorylation of Thr495eNOS, which is similar to the effect of Go6976. Mat could also increase the expression of Ser1177eNOS and when combined with Go6976 has no significant effect on this regulatory effect, but was significantly decreased when combined with LY294002 (Figure 8D and E).

## 4 DISCUSSION

Obesity is a chronic metabolic disorder that is associated with numerous diseases, including hyperlipidemia, diabetes mellitus, hypertension, atherosclerosis, cardiovascular disease and cancer.^26^, ^27^, ^28^, ^29^ Abnormal metabolism of blood lipids caused by an HFD is the main cause of cardiovascular disease and increased plasma LDL levels are regarded as a major risk factor. During oxidative stress, LDLs are oxidized at the vessel wall, thereby resulting in ox‐LDL, which can cause endothelial dysfunction and is considered as an early and critical step in atherogenesis.^30^, ^31^, ^32^, ^33^ Thus, lipid‐lowering therapies are an effective method to improve HFD‐induced vascular injury. The present study showed that consumption of an HFD in mice significantly increased body weight and body fat compared to a regular diet. In addition, blood lipid levels also significantly increased with the consumption of an HFD. However, after treatment with Mat, the plasma TC, TG and LDL levels of HFD‐fed mice significantly decreased and HDL levels also normalized. Lipid peroxidation in the body can lead to the occurrence of inflammatory reactions. Compared to mice fed a normal diet, the serum TNF‐α and IL‐6 levels of mice fed with the HFD significantly increased, whereas IL‐10 levels decreased. In contrast, the serum levels of the inflammatory factors TNF‐α and IL‐6 significantly decreased and IL‐10 levels increased after Mat treatment of mice fed with the HFD. Endothelial cells are involved in regulating the proliferation of vascular smooth muscle cells. Endothelial injury can cause abnormal proliferation of vascular smooth muscles and can increase the thickness of vascular walls.^14^, ^34^, ^35^ Histological analyses showed that aorta wall thickness was significantly augmented in the model mice and pretreatment with Mat significantly reduced the thickening of blood vessel walls.

The integrity of endothelial function plays an important role in the occurrence of cardiovascular disease.^36^ ox‐LDL is an important factor in endothelial injury.^37^ To further confirm the protection mechanism of Mat in endothelial injury, the ox‐LDL‐induced HUVEC injury model was utilized. Cell viability of HUVECs significantly decreased after the administration of ox‐LDL. LDH, which is a marker for cell damage, also increased in model cells. Pretreatment with Mat resulted in normal cell viability and LDH levels. NO is an important cardiovascular regulating factor that not only expands blood vessels but also inhibits platelet adhesion and aggregation, activates leucocyte chemotaxis, induces the proliferation and migration of vascular smooth muscle cells and reduces lipid peroxidation damage of endothelial cells, which were induced by ox‐LDL. NO production in endothelial cells relies on eNOS activity. The increase in eNOS activity enhances endothelial NO synthesis. Our results show that Mat could significantly increase NO levels and eNOS activity and attenuated intracellular ROS production in ox‐LDL‐induced HUVEC injury. ROS generated by ox‐LDL elicits endothelial apoptosis. Mat could also decrease apoptosis of HUVECs when treated with ox‐LDL. The PI3K/Akt signalling pathway plays a crucial role in endothelial dysfunction and cell apoptosis. It has been reported that Akt can activate eNOS and increase NO production. Activation of the PI3K/Akt signalling pathway axis triggers a network of multiple outcomes. PI3K phosphorylates Akt at Ser473, resulting in kinase activation and stimulation of Akt enhances eNOS activity by phosphorylating Ser1177, which in turn increases the release of NO.^38^, ^39^ We investigated the effects of Mat in this regard. The data show that Mat can increase eNOS phosphorylation of Ser1177 and Akt phosphorylation of Ser473 levels. Interestingly, after co‐treatment of Mat with the PI3K inhibitor, the phosphorylation levels of Sre473 Akt and Ser1177 eNOS significantly decreased and there was no significant difference from those of the LY294002 groups. However, Mat could still enhance eNOS activity and increase NO levels after inhibiting the PI3K/Akt pathway through the combination with PI3K inhibitors. Compared to the Mat‐only treatment group, its combination with LY294002 significantly decreased the protective effect of Mat against ox‐LDL‐induced HUVEC injury, but there was still a significant difference compared to the model group. It is worth noting that the protective effect of Mat in HUVEC injury was diminished after the combined application with the eNOS inhibitor. eNOS activity is strongly influenced by a number of post‐translational mechanisms, including protein phosphorylation. eNOS is phosphorylated at several serine residues. S615, S633 and S1177 (human isoform) lie within the two major auto‐inhibitory domains of the C‐terminus in eNOS and work collectively to increase enzyme activity. In contrast to the positive regulatory phosphorylation sites, eNOS is negatively regulated by the phosphorylation of threonine 495(p‐thr495) and phosphorylated eNOS cannot bind to calmodulin (CaM) and thus remains enzymatically inactive.^40^ Our results showed that Mat could inhibit eNOS phosphorylation of Thr495. The role of Mat in mediating eNOS Thr495 phosphorylation was also observed when treatment was conducted in combination with a PI3K inhibitor. Increased phosphorylation of Thr495 (p‐thr495) is stimulated by activators of PKCα and previous studies have reported that PKCθ and PKCβ are also involved in the regulation of the eNOS pathway and PKCα is mainly involved in the regulation of eNOS495 phosphorylation in ox‐LDL‐treated endothelial cells.^41^, ^42^, ^43^, ^44^, ^45^, ^46^, ^47^ To investigate the affinity of Mat to PKC isoforms, molecular docking studies were performed in this study.^48^ Our results showed that Mat could combine with PKCα and its carbonyl group forms H bonds with the Lys368 amino groups, thereby enhancing intermolecular forces. At the same time, it also induces non‐polar interactions with VAL420, ALA480, VAL353, LYS368 and ALA366, but has low affinity for PKCθ compared to PKCα and a PKCθ inhibitor (sotrastaurin). Therefore, to further prove the regulatory effect of Mat on PKCα, the PKCα inhibitor Go6976 was utilized. Our results showed that the PKCα activity in HUVECs treated with ox‐LDL significantly increased compared to the control, whereas Mat pretreatment effectively decreased the PKCα activity of ox‐LDL‐exposed HUVECs (*P* < 0.05). This inhibitory effect is similar to that of the PKCα inhibitor Go6976. The application of Mat induced a significant increase in eNOS activity and the NO content in ox‐LDL‐induced HUVECs and also decreased the expression of phosphorylation of Ser657 PKCα and the phosphorylation of Thr495eNOS. The PKCα inhibitor had the same effect as that of Mat, but the regulatory effect of Go6976 further improved when combined with Mat. It is important to note that when matrine, LY‐294002 and Go6976 were given at the same time, the regulatory effect did not significantly differ from that of the Go6976‐only treatment group. Furthermore, our results indicated that Mat blocks the phosphorylation of Thr495eNOS, which is similar to the effect of Go6976. Mat could also increase the expression of Ser1177eNOS and its combination with Go6976 did not significant alter its regulatory effect but significantly decreased when combined with LY294002.

In conclusion, Mat effectively improved lipid metabolism, inflammation and the thickness of vascular walls in HFD mice. It also decreased eNOS‐Thr497 phosphorylation and increased eNOS‐S1177 phosphorylation, thereby increasing NO production, which is mediated by PI3K/Akt and PKCα. Our results shed light on the molecular mechanisms underlying the protective effect of Mat in HFD‐induced vascular diseases.

## CONFLICTS OF INTEREST

The authors declare that they have no conflict of interest.
